# Long non-coding RNA *H19* and *MALAT1* gene variants in patients with ischemic stroke in a northern Chinese Han population

**DOI:** 10.1186/s13041-018-0402-7

**Published:** 2018-10-10

**Authors:** Ruixia Zhu, Xu Liu, Zhiyi He

**Affiliations:** Department of Neurology, The First Affiliated Hospital of China Medical University, 155 Nanjing North Street, Shenyang, 110001 China

**Keywords:** Ischemic stroke, Gene variants, H19, MALAT1

## Abstract

Objectives Long non-coding RNAs (lncRNAs) have been identified as key regulators in the development of atherosclerosis, which is a major cause of ischemic stroke. However, to date, there are no reports on the association between lncRNA gene variation and the risk of ischemic stroke. Therefore, we assessed the association between *H19* and *MALAT1* gene polymorphisms and susceptibility to ischemic stroke in a northern Chinese Han population. Methods In our study, we genotyped four genetic variations in lncRNA-*H19* and -*MALAT1* (rs217727, rs2251375, rs619586, and rs3200401) in a case–control study of 567 ischemic stroke patients and 552 control subjects. Results We found that the TT genotype of the rs217727 polymorphism within *H19* was significantly associated with increased risk of ischemic stroke in our northern Chinese Han population (odds ration (OR) = 1.519, 95% confidence interval (CI) = 1.072–2.152, *p* = 0.018). Stratified analysis based on stroke subtype revealed that the increased risk was more evident in small vessel ischemic stroke (OR = 1.941, 95% CI = 1.260–2.992, *p* = 0.02). Individuals with the TT genotype had a 1.941 times higher risk of small vessel ischemic stroke when compared with the subjects of CC + CT. These correlations remained after adjusting for confounding risk factors of stroke (OR = 1.913, 95% CI = 1.221–2.998, *p* = 0.005). However, there was no significant association between *H19* rs2251375 or *MALAT1* rs3200401 and ischemic stroke in either total population analysis or subgroup analysis. Conclusion In conclusion, our findings suggest that the *H19* rs217727 gene polymorphism contributes to small vessel ischemic stroke susceptibility in the Chinese Han population and may serve as a potential indicator for ischemic stroke susceptibility.

## Introduction

Ischemic stroke (IS) is a major threat to health and quality of life in modern society [1]. Studies have identified a series of new candidate gene loci that may contribute to the pathogenesis of IS [2]. In recent years, increasing evidence suggests that new candidate markers long non-coding RNAs (lncRNAs), which are functional RNA molecules that are not translated into proteins, contribute to atherosclerotic-related disease [3]. Research into lncRNAs in IS is just beginning. In light of their abundant expression and strong functions, which have been reported previously, lncRNAs show promise as novel therapeutic targets for IS [4]. Due to the limited window of thrombolytic therapy, the development of new therapies is urgently required.

Non-coding RNA (ncRNA) can be classified into short and long ncRNAs. LncRNAs are a subclass of non-coding RNAs with lengths of more than 200 nucleotides, and lack protein-encoding capacity. They are thought to function through a variety of mechanisms, such as in transcription, translation, genome rearrangement, and chromatin modification [4]. LncRNAs can regulate gene expression at various levels, including through epigenetic, transcriptional, and posttranscriptional regulation [5, 6]. LncRNAs also affect microRNA (miRNA) functions by controlling pre-mRNA splicing or by acting as miRNA sponges. Studies have also suggested that genetic variation in lncRNAs may influence lncRNA expression, the process of splicing, and the stability of mRNA conformation, and thus affect disease susceptibility [7–9]. Notably, previous studies have suggested that miRNA single nucleotide polymorphisms (SNPs) are associated with the development of IS [10]. Due to the various functions of lncRNA and its regulatory role as competing endogenous RNA (ceRNA; or miRNA sponges), it may be reasonable to speculate that lncRNAs directly contribute to the development of IS [11].

Recent studies have demonstrated that lncRNAs are differentially expressed in IS patients, and that the expression of selected lncRNAs change over time after IS [12, 13]. Han et al. [14] identified that *H19* is overexpressed in human atherosclerotic plaques and in rat vascular smooth muscle cells after injury. Furthermore, Wang et al. [15] reported that lncRNA *H19* plays an important role in the process of cerebral I/R injury through its regulation of autophagy activation and apoptosis. Moreover, recent studies also found that polymorphisms in *H19* are associated with risk factors for IS including obesity, birth weight, and blood pressure [16, 17]. We thus speculated that *H19* polymorphisms are associated with IS risk. Recently, the lncRNA metastasis-associated lung adenocarcinoma transcript-1 (*MALAT1*) has been shown to exert a pivotal role in endothelial cell functions and angiogenesis [18]. *MALAT1* is upregulated in endothelial cells in response to hypoxia, and knocking down *MALAT1* promotes endothelial cell migration and inhibits endothelial cells proliferation [19]. In addition, a recent study revealed that *MALAT1* is involved in inflammation and can facilitate the inflammatory cascade by upregulating IL-6 and TNF-a expression [20]. Moreover, endothelial cell dysfunction and inflammation of the vessel wall are thought to be the key events in atherosclerosis progression. This evidence suggests that *MALAT1* and *H19* may play an important role in the pathogenesis of IS.

However, no studies have reported on the association between genetic variants of *H19* and *MALAT1* and the risk of IS. Thus, we genotyped four lncRNA SNPs (*H19*: rs217727, rs2251375; *MALAT1*: rs619586, rs3200401) in a case–control study of 567 IS patients and 552 healthy controls from the Chinese Han population.

## Methods

### Study subjects

A total of 567 IS patients and 552 control subjects were enrolled for this case–control study. All patients were from the Department of Neurology at the First Affiliated Hospital of China Medical University. The rules for inclusion of patients and controls were as described in our previous manuscript [21]. According to TOAST typing, patients were classified into two subtypes: large-artery atherosclerosis (LAA) and small-vessel occlusive (SVO) stroke, while other subtypes were excluded from this study [22]. This study was approved by the ethics committee of the First Affiliated Hospital of China Medical University in accordance with the principles of the Helsinki Declaration. Written informed consent was obtained from all participants.

### SNP selection

Using dbSNP database (https://www.ncbi.nlm.nih.gov/snp/) and Haploview 4.2 software, we selected tagging SNPs within the MALAT1 and H19 gene region according to the criteria of minor allele frequency more than 5% in Chinese Han population. Additionally, linkage disequilibrium value *r2* should be less than 0.8 for the candidate SNP. Moreover, these four well-known lncRNA polymorphisms (rs217727, rs2251375, rs619586, and rs3200401) have been studied and were found to be associated with the risk of various diseases [23–27].These four lncRNA polymorphisms (rs217727, rs2251375, rs619586, and rs3200401) were identified.

### DNA extraction and genotyping

Genomic DNA from each sample was extracted using a DNA blood mini kit, following the manufacturer’s protocol(Promega, Madison, USA). Genotyping of the SNPs (rs217727, rs2251375, rs619586, and rs3200401) was carried out using the polymerase chain reaction–ligase detection reaction (PCR-LDR) method. The sequences of primers for PCR are shown in Table 1.The PCR programs were carried out with a total volume of 10 μl, including 1× GC-I buffer, 3.0 mM Mg^2+^, 0.3 mM dNTPs, 1 U Hot-Start Taq polymerase (Qiagen Inc), 1 μl genomic DNA, and 1 μl of each primer. The cycling parameters were as follows: 95 °C for 2 min; 11 cycles of 94 °C for 20 s, 65 °C for 40 s, and 72 °C for 90 s; 24 cycles at 94 °C for 20 s, 59 °C for 30 s, and 72 °C for 90 s; and a final extension step at 72 °C for 2 min. The following LDR was carried out with a final volume of 10 μl, containing 1 μl 10× ligation buffer, 2 μl PCR product, 6 μl double-distilled H_2_O, 0.4 μl of each discriminating probe, and 0.25 μl Taq DNA ligase. The LDR parameters were as follows: 38 cycles of 94 °C for 60 s and 56 °C for 4 min. After the reaction, the LDR product was then analyzed using the ABI 3730XL DNA Sequencer. About 5% of the samples were randomly selected for repeated genotyping. Repeatability of results was 100%.

**Table 1:** Primer sequences and probes for LDR of *H19* and *MALAT1*

SNPs	Primer sequences and probes
rs217727F	CCGTCTCCACAACTCCAACCAG
rs217727FA	TACGGTTATTCGGGCTCCTGTGGCTGGTGGTCAACCGTGCA
rs217727FG	TTCCGCGTTCGGACTGATATGGCTGGTGGTCAACCGTACG
rs217727FP	CCRCAGGGGGTGGCCRTGAATTTTTTTTTT
rs217727R	CCAGACCTCATCAGCCCAACAT
rs2251375F	CCAGCGCCCTGCACATACTT
rs2251375FA	TGTTCGTGGGCCGGATTAGTGCCACCATCACGGCTCACAA
rs2251375FC	TCTCTCGGGTCAATTCGTCCTTGCCACCATCACGGCTCACAC
rs2251375FP	CTCACGTTCCTGGAGAGTAGGGGTTTTTTTTTT
rs2251375R	AGCACAAGCTCGGTCAACTGG
rs3200401F	CACAGAGAATGCAGTTGTCTTGACTTC
rs3200401R	ACTCCAAGCATTGGGGAACACA
rs3200401RC	TTCCGCGTTCGGACTGATATGCATTTACTTGCCAACAGAACAGAGAG
rs3200401RP	ACCTGAAGTCAAGACAACTGCATTCTTTTTTTTTTTTTTTT
rs3200401RT	TACGGTTATTCGGGCTCCTGTGCATTTACTTGCCAACAGAACAGAGAA
rs619586F	GCAAGCAGTTGGGGGAGAAAGT
rs619586R	CGGGTCATCAAACACCTCACAA
rs619586RA	TGTTCGTGGGCCGGATTAGTCACTTCTTGTGTTCTCTTGAGGGACTGT
rs619586RG	TCTCTCGGGTCAATTCGTCCTTCACTTCTTGTGTTCTCTTGAGGGACTGC
rs619586RP	AGGTATAGTTTACCACCTTTTGAAGGAAGATTTTTTTTTTTTTTTT

### Statistical analysis

Differences in the distribution of demographic variables and genotypes were evaluated by Pearson’s χ2 test or the Student’s t test. The Hardy–Weinberg equilibrium was evaluated by the χ2 test for genotypes in two groups. The odds ratio (OR) and 95% confidence interval (CI) from logistic regression analyses were calculated to estimate the association between lncRNA polymorphisms and risk of IS. Genotype frequencies were compared between cases and controls under the additive model, dominant model, and recessive model. A *p* value of less than 0.05 for two-sided was considered statistically significant. All analyses were conducted with SPSS 16.0 software.

## Results

The characteristics of cases and controls are presented in Table 2. There was no significant difference between IS patients and controls in age (61.9 ± 9.52 vs. 61.7 ± 10.17) and gender (male 64.2% vs. 63%). However, the prevalence of conventional risk factors for IS, such as drinking, smoking, hypertension, diabetes mellitus, and hyperlipidemia was significantly greater in the IS patient group than in the control group.

**Table 2:** Clinical characteristics of the case and control

	Case (*n* = 567)	Control (*n* = 552)	*P* value
Age(mean ± SD)	61.72 ± 10.17	61.9 ± 9.52	0.677
Male gender, *n* (%)	364 (64.2%)	348 (63.0%)	0.668
Hypertension, *n* (%)	396 (69.8%)	320 (57.9%)	0.000
Diabetes, *n* (%)	213 (37.5%)	124 (22.3%)	0.000
Hypercholesterolemia,*n* (%)	259 (45.7%)	204 (36.9%)	0.003
Smoking, *n* (%)	204 (35.9%)	107 (19.4%)	0.000
Alcohol drinker (%)	113 (19.9%)	65 (11.8%)	0.000
BMI (Kg/m2)	25.05 ± 3.70	25.34 ± 3.30	0.285

All of the polymorphism frequencies followed the Hardy–Weinberg equilibrium (*p* = 0.284 for rs3200401, *p* = 0.137 for rs619586, *p* = 0.064 for rs217727, and *p* = 0.76 for rs2251375) in 552 healthy controls. The frequencies of CC, CT, and TT genotypes for the rs217727 polymorphism in patients were 38.4, 45.8 and 15.9%, while in controls, the values were 39.3, 49.6, and 11.1%, respectively. The frequencies of the rs217727 C and T alleles were 61.2 and 38.7% in patients and 64.1 and 35.7% in controls, respectively. In the *H19* gene rs217727 polymorphism, the TT genotype was associated with an increased risk of IS both in the recessive genetic model (OR = 1.519, 95% CI =1.072–2.152, *p* = 0.018) and additive model (OR = 1.479, 95% CI = 1.009–2.138, *p* = 0.044), as shown in Table 3. That is, TT genotype carriers of rs217727 had a 1.479-fold increased risk of IS compared with C allele carriers. The frequencies of the CC, CA, and AA genotypes of rs2251375 were 31, 49.2, and 19.8% in the IS patients and 32.2, 49.6, and 18.1% in the controls, respectively. However, there were no significant differences between IS patients and controls in the genotype and allele frequencies of rs2251375 (Table 4). In addition, the distributions of the *MALAT1* rs3200401, rs619586 genotypes and alleles were similar between IS patients and healthy controls (Tables 5, 6). Because the frequency of GG genotype is lower, the rs619586 polymorphism was excluded in further subgroup analysis.

**Table 3:** Allele and genotype frequencies of H19 rs217727 for IS patients and the control group

	Case	Control	*p*	OR	95%CI
Genotype					
CC	218	217	Reference		
CT	259	274	0.638	0.941	0.73–1.212
TT	90	61	0.044	1.469	1.009–2.138
Dominant effect					
CT + TT vs CC	349/218	335/217	0.767	1.037	0.815–1.319
Recessive effect					
TT vs CT + CC	90/477	61/491	0.018	1.519	1.072–2.152
Allele					
T	439	396	Reference		
C	695	708	0.164	1.129	0.951–1.341

**Table 4:** Allele and genotype frequencies of H19 rs2251375 for the patients with IS and the control group

	Case	Control	*p*	OR	95%CI
Genotype					
CC	176	178	Reference		
CA	279	274	0.829	1.030	0.789–1.345
AA	112	100	0.473	1.133	0.806–1.593
Dominant effect					
CA + AA vs CC	391/176	374/178	0.665	1.057	0.822–1.360
Recessive effect					
AA vs CA + CC	112/455	100/452	0.485	1.133	0.825–1.501
Allele					
A	503	474	Reference		
C	631	630	0.498	1.059	0.896–1.252

**Table 5:** Allele and genotype frequencies of MALAT1 rs3200401 for the patients with IS and the control group

	Case	Control	*p*	OR	95%CI
Genotype					
CC	406	397	Reference		
CT	150	146	0.973	1.005	0.77–1.312
TT	11	9	0.695	1.195	0.649–2.915
Dominant effect					
CT + TT vs CC	161/406	155/397	0.907	1.016	0.783–1.318
Recessive effect					
TT vs CT + CC	11/556	9/543	0.696	1.194	0.491–2.903
Allele					
T	172	164	Reference		
C	962	940	0.836	1.025	0.813–1.292

**Table 6:** Genotype and allele distributions of MATAL1 rs619586 for the patients with IS and the control group

	Case	Control	*p*	OR	95%CI
Genotype					
AA	465	464	Reference		
AG	97	87	0.509	1.113	0.811–1.527
GG	5	1	0.104	4.989	0.581–42.87
Dominant effect					
GG + AG vs AA	102/465	88/464	0.362	1.157	0.846–1.581
Recessive effect					
GG vs AA+AG	5/562	1/551	0.109	4.902	0.571–42.094
Allele					
G	107	89	Reference		
A	1027	1015	0.25	1.188	0.885–1.595

We then performed stratified analyses to examine the effects of lncRNA SNPs on the risk of IS according to stroke subtype. We further divided the stroke group into two subgroups (LAA and SVO) according to the TOAST classification. This study included 211 SVO stroke patients, 356 LAA stroke patients, and 552 controls. The TT genotype of *H19* rs217727 was associated with significantly increased SVO stroke risk compared with the CC + CT genotype (Table 7). In the LAA stroke subgroup, there were no significant differences in either the genotypic distribution or the allelic frequency between patients and controls for rs217727. When analyzing rs2251375, we again did not find a significant association between *H19* rs2251375 and IS in either the LAA or SVO subgroup (Table 8). Similar to rs2251375, subgroup analysis based on stroke subtype revealed that *MALAT1* rs3200401 was not significantly associated with IS risk in all genetic models (Table 9).

**Table 7:** Allele and genotype frequencies of H19 rs217727 and its relationship with stroke subtypes

	LAA					SVO				
	Case	Control	*P*	OR	95%CI	Case	Control	*P*	OR	95%CI
Genotype										
CC	139	217	Reference			79	217	Reference		
CT	168	274	0.765	0.957	0.719–1.275	91	274	0.607	0.912	0.643–1.295
TT	49	61	0.304	1.254	0.814–1.932	41	61	0.010	1.846	1.151–2.961
Dominant effect										
CT + TT vs CC	217/139	335/217	0.936	1.011		132/79	335/217	0.635	1.082	0.780–1.501
Recessive effect										
TT vs CT + CC	49/307	61/491	0.221	1.285	0.77–1.329	41/170	61/491	0.02	1.941	1.260–2.992
Allele										
T	266	396	Reference		0.859–1.921	173	396	Reference		
C	446	708	0.520	1.066	0.877–1.296	249	708	0.064	1.242	0.987–1.563

**Table 8:** Allele and genotype frequencies of H19 rs2251375 and its relationship with stroke subtypes

	LAA					SVO				
	Case	Control	*P*	OR	95%CI	Case	Control	*P*	OR	95%CI
Genotype										
CC	115	78	Reference			61	178	Reference		
CA	179	274	0.942	1.011	0.740–1.366	100	274	0.739	1.065	0.736–1.542
AA	62	100	0.838	0.960	0.647–1.423	50	100	0.097	1.459	0.935–2.281
Dominant effect										
CA + AA vs CC	241/115	374/178	0.986	0.997	0.75–1.326	150/61	374/178	0.374	1.17	0.827–1.656
Recessive effect										
AA vs CA + CC	62/294	100/452	0.788	0.953	0.672–1.352	50/161	100/452	0.083	1.404	0.956–2.061
Allele										
A	303	474	Reference			200	474			
C	409	630	0.874	0.985	0.814–1.191	222	630	0.117	1.197	0.956–1.50

**Table 9:** Allele and genotype frequencies of MALAT1 rs3200401 and its relationship with stroke subtypes

	LAA					SVO				
	Case	Control	*P*	OR	95%CI	Case	Control	*P*	OR	95%CI
Genotype										
CC	252	397	Reference			154	397	Reference		
CT	97	146	0.767	1.047	0.774–1.415	53	146	0.722	0.936	0.649–1.348
TT	7	9	0.690	1.225	0.451–3.32	4	9	0.823	1.146	0.348–3.775
Dominant effect										
CT + TT vs CC	104/252	155/397	0.712	1.057	0.788–1.419	57/154	155/397	0.769	0.948	0.664–1.354
Recessive effect										
TT vs CT + CC	7/349	9/543	0.707	1.21		4/207	9/543	0.8	1.166	0.355–3.827
Allele										
T	111	164			0.477–3.279	61	164			
C	601	940	0.670	1.059	0.815–1.375	361	940	0.844	0.969	0.705–1.351

Multivariate logistic regression analysis was used to evaluate associations between *H19* rs217727 and IS risk. After adjusting for confounding factors including drinking, smoking, hypertension, diabetes mellitus, and hyperlipidemia, the impact of TT genotype on IS risk was still remarkable (OR = 1.515, 95% CI = 1.055–2.177), as shown in Table 10. The TT genotype of rs217727 was also significantly associated with an increased risk of IS compared with the TT genotype in the SVO subgroup (OR = 1.913, 95% CI = 1.221–2.998), as shown in Table 11.

**Table 10:** Ischemic stroke risk factors in the logistic regression analysis

	*p*	OR	95%CI
Hypertension	0.003	1.474	1.139–1.907
Diabetes	0.000	1.893	1.439–2.490
Hypercholesterolemia	0.029	1.322	1.028–1.700
Smoking	0.000	2.158	1.599–2.913
Genotype TT of H19 rs217727	0.025	1.515	1.055–2.177

**Table 11:** Ischemic stroke risk factors of small vessel ischemic stroke in the logistic regression analysis

	*p*	OR	95%CI
Hypertension	0.029	1.489	1.042–2.128
Diabetes	0.007	1.647	1.145–2.369
Hypercholesterolemia	0.005	1.608	1.152–2.244
Smoking	0.000	1.995	1.361–2.926
Genotype TT of H19 rs217727	0.005	1.913	1.211–2.998

## Discussion

To the best of our knowledge, this is the first study to investigate the associations between *H19* gene polymorphisms (rs217727 and rs2251375), *MALAT1* gene polymorphisms (rs619586 and rs3200401), and IS susceptibility in the northern Chinese Han population. Our results suggested that the TT genotype of the *H19* gene rs217727 polymorphism was associated with an increased risk of IS. Additionally, we identified a more prominent risk effect of the rs217727 TT genotype in the SVO stroke subgroup. In contrast, the *H19* SNP rs2251375 and the *MALAT1* SNP rs3200401 were not related to IS susceptibility.

Several studies have shown that the *H19* gene rs217727 polymorphism is associated with disease susceptibility. Yang et al. [23] identified that the *H19* gene rs217727 and rs2839698 polymorphisms are associated with increased gastric cancer risk in a Chinese Han population. In addition, Verhaegh et al. [24] reported that a genetic variant of *H19* was associated with a decreased risk of bladder cancer in European Caucasians. Furthermore, a study by Gao et al. [25] showed that rs217727 polymorphisms of *H19* are associated with the risk and severity of CAD in a Chinese population. To the best of our knowledge, although the relationship of lncRNA *H19* expression with some cancers has been confirmed, an association of *H19* lncRNA with IS has not been reported.

In our study, we observed that the TT genotype of the rs217727 polymorphism was associated with an increased risk of IS. A 52% increased risk of IS was identified in IS patients with TT genotype within rs217727 compared with C allele carriers (OR = 1.519, 95% CI = 1.072–2.152), indicating that the T allele might be a risk factor for IS. Logistic regression analysis demonstrated that the TT genotype of rs217727 was independently correlated with an increased risk of IS, even after adjusting for confounding risk factors. However, the rs2251375 polymorphism showed no significant association with IS disease. To further assess the risk of this lncRNA polymorphism for IS, a stratified analysis was performed using subgroups of stroke subtypes. The increased risk of IS for the rs217727 polymorphism TT genotype was more evident in the SVO stroke subgroup. Subjects with the TT genotype had a 1.941 times higher risk of having an SVO stroke compared with the subjects of CC + CT genotypes (*p* = 0.02, OR = 1.941, 95% CI = 1.260–2.992). Moreover, individuals carrying a T allele of rs217727 exhibited larger likelihood in getting SVO stroke. The rs217727 TT genotype remained a risk factor for IS even after adjustment for confounding factors (OR = 1.913, 95% CI =1.221–2.998).However, stratified analyses revealed no significant association between rs2251375 and IS in either the LAA or the SVO stroke subgroup.

Growing evidence indicates that a large number of lncRNAs can serve as miRNA “sponges” by sharing common MREs (miRNA Response Elements), influencing post-transcriptional regulation by inhibiting available miRNA activity [28]. However, the rs217727 polymorphism did not combine with miRNA using the lncRNASNP database. Thus, on the basis of current information, we speculate that genetic variants of lncRNA may alter its structure and expression level, ultimately contributing to IS susceptibility [8, 9]. Additionally, the rs217727 polymorphism may alter translational efficiency and mRNA conformation, which may ultimately influence RNA–mRNA interactions and RNA–protein interactions [7] Thus, it is likely that the rs217727 polymorphism affects the onset of IS through regulation of lncRNA *H19* expression. The precise mechanism of *H19*’s role in IS susceptibility remains unclear, however, and further studies are required to verify our hypothesis.

*MALAT1* is closely related to endothelium function in atherosclerosis through the regulation of endothelial cell proliferation and migration [19]. Furthermore, *MALAT1* plays an essential role in macro- and micro-vascular angiogenesis by regulating endothelial cells in stressful conditions. *MALAT1* is involved not only in angiogenesis but also in inflammation [29]. LncRNA-*MALAT1* promotes an inflammatory response through activating serum amyloid A3 protein [19]. This evidence suggested that *MALAT1* may participate in the pathogenesis of IS; however, our study did not demonstrated any significant association of *MALAT1* gene rs3200401 polymorphism with IS in any genetic model in the general population. We also found no significant association between them in a subgroup analysis of stroke subtype. Moreover, we still found no correlation between *MALAT1* gene rs619586 polymorphism and ischemic stroke risk. Because the frequency of GG genotype is lowest in our study (0.9% in the IS patients and 0.2% in the controls), we excluded this SNP in further subgroup analysis. Thus, a larger sample size is required to confirm the role of rs619586 in IS risk in the future.

To date, this is the first report about the association of lncRNA SNPs and IS susceptibility. In summary, our findings indicate that the *H19* gene rs217727 polymorphism contributes to the susceptibility of small vessel IS in the northern Chinese Han population, and may serve as a novel lncRNA target for IS susceptibility. Functional analysis of lncRNAs with respect to stroke pathophysiology is just beginning. Moreover, a broader perspective of miRNA–lncRNA–mRNA interactions will be important for constructing IS disease-specific networks in the future.

## Abbreviations

CI: Confidence interval
IS: Ischemic stroke
LAA: Large-artery atherosclerosis
lncRNAs: Long non-coding RNAs
MALAT1: Metastasis-associated lung adenocarcinoma transcript-1
MRE: miRNA Response Elements
OR: Odds ratio
SNP: Single nucleotide polymorphisms
SVO: Small-vessel occlusive stroke
